# The effects of antifungal therapy on the recurrence of aspergillus infection after pulmonary aspergilloma resection: a study protocol for a single-center, prospective, non-blind, randomized, 24-month, parallel group study

**DOI:** 10.1186/s12890-021-01702-4

**Published:** 2021-10-29

**Authors:** Xianqiu Chen, Yiming Zhou, Lijuan Zhang, Jinfu Xu, Shuo Liang

**Affiliations:** 1 Department of Respiratory and Critical Care Medicine, Shanghai Pulmonary Hospital, School of Medicine, Tongji University, 507 Zheng Min Road, Shanghai, 200433 China; 2 Department of Thoracic Surgery, Shanghai Pulmonary Hospital, School of Medicine, Tongji University, Shanghai, 200433 China; 3 Department of Epidemiology, School of Medicine, Tongji University, Shanghai, 200092 China

**Keywords:** Antifungal therapy, Aspergilloma, Safety, Recurrence

## Abstract

**Background:**

In recent years, the incidence of pulmonary aspergilloma has increased. The harm of aspergilloma is life-threatening massive hemoptysis, and the conventional treatment is surgical treatment. However, whether the antifungal treatment after surgery is required and the course of treatment before and after surgery are still unclear.

**Methods:**

In this study, patients with pulmonary aspergilloma confirmed pathologically after surgery will be selected as subjects to conduct a single-center, randomized, parallel grouping, prospective, 2-year clinical study. Through regular visits, the recurrence of aspergillus infection, quality of life, lung function indicators, safety of antifungal therapy and other indicators were recorded to evaluate the recurrence risk of aspergillus infection and safety of antifungal agents. Cox proportional risk regression model was used to analyze the influencing factors of antifungal therapy on aspergillus infection recurrence after aspergillus bulbectomy. Cox multiple regression model was used for optimal model fitting, and regression coefficient (β), relative risk (RR) and 95% confidence interval of RR were calculated.

**Discussion:**

The study will explore whether antifungal therapy could improve the quality of life, reduce the recurrence of aspergillus infection, and ultimately improve the prognosis of patients with aspergilloma. The study results will provide high-quality evidence-based medical evidence for the formulation, revision and optimization of international and domestic clinical guidelines and expert consensus on chronic aspergillus lung disease, effectively improve the clinical treatment effect of aspergilloma, and form the latest concept of diagnosis and treatment of aspergilloma.

*Trial registration*: The trial was registered on the Chinese Clinical Trial Registry website (https://www.chictr.org.cn/showprojen.aspx?proj=33231). Registration number: ChiCTR1800019990.

## Background

Pulmonary aspergilloma is a kind of aspergillus parasitic in the lung cavity, mycelium and cell debris inside the hole to form a sphere. It most commonly occurs in the existing pulmonary cavity, including tuberculosis, lung abscess, bronchiectasis, pulmonary cyst, cytoplasmic disease, lung cancer and other diseases of pulmonary cavity formation, which is one of the lung intractable infectious diseases. The harm of aspergilloma is life-threatening massive hemoptysis. Currently, there is no consensus on the best treatment of aspergilloma, and there is a lack of double-blind, randomized, placebo-controlled clinical studies on its treatment. Information on treatment comes mainly from case reports and retrospective studies. In general, patients with asymptomatic aspergilloma do not need special treatment and can be observed regularly [1], and treatment is only considered when patients have symptoms of hemoptysis [2]. Some scholars believe that surgery is the best treatment [3, 4]. As for the acute and long-term prognosis of patients with pulmonary aspergilloma treated by surgery, studies [5] showed that the overall survival rate at 2, 5 and 10 years was 86.6%, 79.4% and 79.4%, respectively, and the disease-free survival rates were 86.6%, 72.6% and 72.6%, respectively. The survival rate may be affected by the absence of antifungal treatment, postoperative pulmonary dysfunction and recurrence of fungal infection. These questions need to be further explored in clinical studies. This study was designed to evaluate the difference in recurrence risk of aspergillus infection and safety of antifungal agents in patients with pathologically confirmed aspergilloma after surgery in a single-center, randomized, parallel grouping, prospective, 2-year clinical study.

## Methods

### Study design and setting

The study was a single-center, prospective, non-blind, randomized, 24-month parallel controlled trial. The data of patients diagnosed with aspergilloma and surgically excision from Shanghai Pulmonary Hospital from January 1, 2020 to December 31, 2022 will be collected. After providing informed consent, enrolled patients will be randomly assigned for study group or the control group at a ratio of 1:1 using the block randomization method. Each patient will then receive a numeric randomization code. According to the literature and the results of the preliminary experiment, the recurrence rate of the experimental group is expected to be less than 1%, and that of the control group is about 20%. According to the ɑ value of 0.05 and the degree of certainty of 0.8, the sample content of each group is estimated by PASS 15 software to be 37 people, with the amplification of 1.1 times, and 40 patients are planned to be included in each group.

**Fig. 1:**
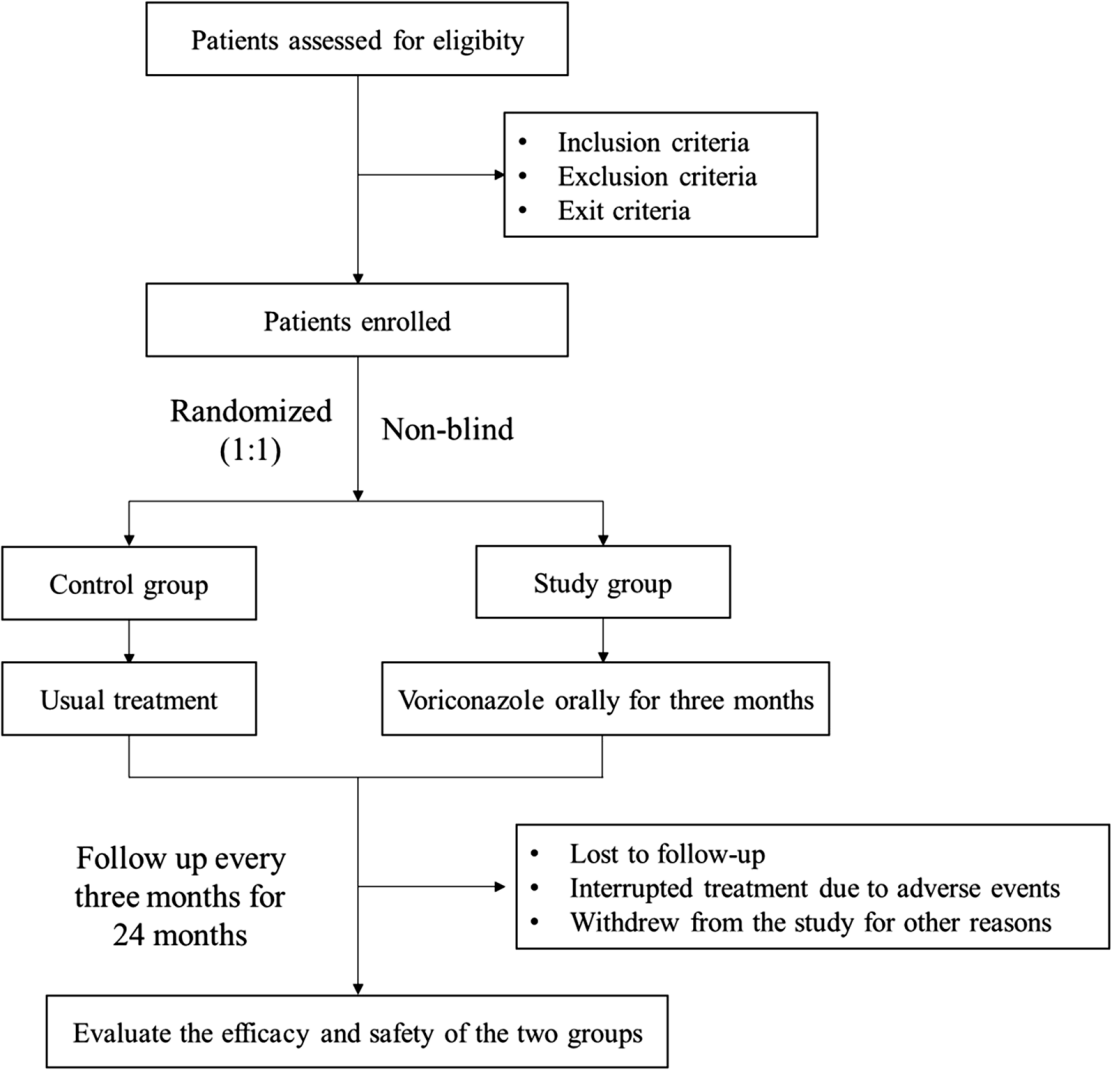
Research process and flow chart

The study group will receive voriconazole orally for three months, twice a day, 0.2 g each time. The control group will not receive antifungal therapy. Detailed research process is described in Fig. 1.

### Inclusion criteria

All the following criteria must be met: (1) Aspergillomas are in the lung. (2) Patients with clear pathological diagnosis of aspergilloma and surgical resection. (3) Written informed consent is signed.

### Exclusion criteria

(1) Patients with a severe disease other than the lung that, in the investigator’s judgment, would put the subject at risk for participating in the study or affect the results of the study and the subject’s ability to participate in the study. (2) Patients with a history of serious heart disease, such as acute myocardial infarction, congestive heart failure (NYHA cardiac function grade III and above), severe arrhythmia and other acute heart disease. (3) Serious primary diseases of important organs and systems, such as acute stroke, moderate or above hypertension after treatment, active gastric ulcer, diabetes (with serious complications), malignant tumor, etc. (4) Patients with confirmed and suspected lung cancer. (5) Limited understanding ability and poor compliance. (6) No legal capacity or limited legal capacity. (7) Those who participated in clinical trials of other drugs within 30 days before screening and did not reach the end point. (8) Pregnant and lactating women and women of childbearing age who do not agree to use effective contraceptive measures during the study. (9) Persons with mental or physical disabilities. (10) Suspected or true history of alcohol and drug abuse. (11) Known intolerance to drug treatment. (12) Aspartate aminotransferase (AST) and Alanine transaminase (ALT) three times higher than the upper limit of normal, creatinine more than 176.8 mmol/L. (13) Shock or other hemodynamic instability. (14) Those who are in the active stage of infectious diseases such as hepatitis A, hepatitis B, Acquired Immune Deficiency Syndrome (AIDS), tuberculosis and connective tissue diseases. (15) The researcher considers it inappropriate to participate in this study.

### Exit criteria

1. Withdrawal decided by the researcher:
  (a) Refers to the decision of the investigator to withdraw an enrolled subject from the study if it is not suitable to continue the study during the study. (b) Subjects have poor compliance with the protocol. (c) Severe adverse events or intolerable adverse events occur during the study. (d) The subject has some comorbidities, complications or special physiological changes and is not suitable for further study. (e) Other circumstances in which the investigator determines the need to withdraw from the study.
2. Subjects voluntarily withdraw from the study:
  (a) The subject is unwilling to continue the clinical trial and proposes withdrawal to the investigator. (b) lost to follow-up. (c) Subjects who withdraw early shall indicate the reasons for withdrawal on the case report form. Patients who withdraw due to adverse events should be followed up until the adverse events disappear or have a clear explanation.

### Elimination criteria

Prior to statistical analysis of the data, the main researcher, the sponsor and the statistical unit shall judge whether to exclude the individual cases. If one of the following situations occurs, the principal investigator shall comprehensively judge whether to exclude the subject based on the degree of completion of the test and the reasons for withdrawal, and make relevant explanations:

1. The selection of individual subjects violates the inclusion/exclusion criteria and should not be included in the test;
2. During the study period, the researcher considers that the subject has other factors that can not continue to participate in the study, and actually discontinues the subject’s continued participation in the study;
3. During the test, the subjects do not comply with the test plan and the compliance is poor. For example, the samples for effectiveness and safety evaluation cannot be collected as required by the test plan, and there is no data, etc.
  The Investigator has the right to discontinue the subject’s participation in the study if: the investigator must carefully evaluate the discontinued subject, take active treatment, and record this procedure in detail in the original notebook. If a subject withdraws from the study due to an adverse event, abnormal laboratory test values, or test failure, he or she must also be assessed and treated accordingly, and this procedure must be documented in detail in the original notebook. The investigator may also determine at any time during the study whether or not the subject should continue the clinical trial based on medical judgment.
4. Termination test standard:
  Termination of a trial is when the trial has not completed the evaluation of all subjects as planned. After cessation of the study, new subjects will not be included, and those enrolled but not yet out of the study will be examined and interviewed in accordance with the investigator’s consultation. If the study is found to have no clinical value during the trial, the trial should be terminated;
  (a) Major errors in the clinical trial protocol were found during the trial, making it difficult to evaluate the effect of the study. (b) If serious deviations occur in the implementation of the test, the test should be stopped if it is difficult to evaluate the effect of the study if it continues. (c) Termination at the request of the investigator (for reasons of funding, management, etc.). (d) The China Food and Drug Administration orders the termination of the trial for some reason. (e) The ethics committee shall terminate the trial from the perspective of protecting the rights and interests of the subjects.

For all subjects who terminate the study early, the investigator should obtain, as far as possible, the reason for withdrawal from the study, such as adverse events, ineffectiveness of corrective actions, withdrawal based on the investigator’s decision, or other reasons, and the reason for withdrawal should be recorded in the case report form (CRF). Subjects who quit early should complete the early exit interview according to the schedule of the visit procedure.

### Study endpoints

(1) Primary end point: Recurrence of Aspergillus infection. (2) Secondary end point: days of antifungal treatment. (3) Safety evaluation index: side effects of antifungal treatment.

### Clinical data to be collected

1. Basic information:
  (a) Name, sex and age. (b) Living and working environment. (c) complications.
2. Treatment after aspergilloma resection:
  (a) Preoperative serological indexes of aspergillus infection: 1,3-β -D glucan detection (G test) and galactose mannitolglycan antigen detection (GM test). (b) Serological indexes of postoperative aspergillus infection: G test and GM test. (c) Preoperative blood routine biochemical indexes: liver function, kidney function and electrolyte. (d) Postoperative biochemical indexes: liver function, kidney function and electrolyte. (e) Preoperative immune indicators: CD4, CD8, CD4/CD8, immune globulin (Ig) A, IgG, IgM in peripheral blood. (f) Postoperative immune indicators: CD4, CD8, CD4/CD, IgA, IgG, IgM in peripheral blood. (g) Whether it is antifungal therapy, antifungal drug, dose and course of treatment. (h) Chest computerized tomography (CT). (i) The time and results of fungal smear and culture of lower respiratory tract specimens.

### Research indicators

(1) Laboratory examination. (2) Imaging examination. (3) Surgical method. (4) Treatment.

### Prognosis assessment

Telephone and outpatient follow-up.

### Information recorded during follow-up

General condition, quality of life score [St. George’s Respiratory Questionnaire (SGRQ) score], lung function, sputum culture, G and GM test of peripheral blood, chest CT, immune status, etc. were recorded in baseline. Quality of life score, sputum fungal culture, G and GM test of peripheral blood, chest CT, and immune status were interviewed every three months. Checklist for clinical data collection and follow-up plan of enrolled patients is showed in Table 1.

**Table 1:** Checklist for clinical data collection and follow-up plan of enrolled patients

	Screening stage	Treatment period (one day to three months after enrollment)	Evaluation period (once every three months for 24 months)
Informed consent	X		
Demographic statistics	X		
History of present illness and treatment	X		
Symptoms and vital signs	X	X	X
Inclusion/exclusion criteria	X		
Blood routine	X	X	X
Blood biochemical test	X	X	X
G test of peripheral blood	X	X	X
GM test peripheral blood	X	X	X
CD4/CD8	X	X	X
IgA, IgG, IgM	X	X	X
HCG of peripheral blood or urine	X		
Noninvasive oxygen saturation	X		
Chest CT	X	X	X
Electrocardiograph	X	X	X
Fungal smear and culture	X	X	X
Basic drugs and combined drugs	X	X	X
Distribution of research drugs	X		
Recall of research drug		X	X
Evaluation of adverse events	X	X	X
Schedule of next visit		X	X
Compliance evaluation		X	X
effectiveness evaluation		X	X
SGRQ score	X	X	X
Evaluation of microbiological efficacy		X	X
Survival		X	X

HCG, human chorionic gonadotropin; Ig immune globulin; SGRQ, St. George’s Respiratory Questionnaire

### Statistical analysis

Double entry of data was performed with Epidata 3.1 software, and data analysis was performed with SAS 9.4 (SAS Institute, Cary, North Carolina) statistical software. Before the analysis, normality test was carried out, and the normalized data were consistent with normality. Mean ± standard deviation was used for continuous variables, and percentage was used for classified variables. The baseline data were analyzed by T test or Fisher’s exact probability, chi-square test, etc. Cox proportional risk regression model was used to analyze the influencing factors of antifungal therapy on aspergillus infection recurrence after aspergillus bulbectomy. Cox multiple regression model was used for optimal model fitting, and regression coefficient (β), relative risk (RR) and 95% confidence interval (CI) of RR were calculated.

### Safety indicators

Including recurrence of aspergillus infection and adverse reactions such as liver and kidney function damage and etc.

### Risk control

The risk of patients participating in this study comes from the risk of the disease itself. Theoretically, the study itself does not increase the risk of disease in patients. Patients may still discontinue treatment or withdraw from the study at any time, and subjects may discontinue the study at any time or for any reason at their own request or at the request of the Investigator. For all subjects who withdraw from the study for any reason, contact them or their family or healthcare provider 24 weeks after withdrawal to determine the subject’s life status. Subjects may voluntarily discontinue treatment at any time without affecting further treatment.

### Trial status

The study is ongoing. The protocol was approved on the 24 March 2021 by the Ethics Committee of Shanghai Pulmonary Hospital, Tongji University. The trial was opened for recruitment on 1 April 2021; the first patient was enrolled on 20 April 2021 and randomized on 21 April 2021. A total of 8 patients have been enrolled at present.

### Dissemination plans

The results will be published in high-quality peer-reviewed journals at the end of study.

## Discussion

Recent years, with the increasing incidence of pulmonary tuberculosis and the widespread use of immunosuppressive agents and hormones, pulmonary aspergillomas have increased year by year. Pulmonary aspergillomas themselves are wrapped around aspergillus silk. Aspergillus grows in cave walls and tends to invade local structures, especially blood vessels. In some cases, aspergillomas can alter their benign chronic processes to become invasive or even fatal.

At present, the treatment of aspergilloma is not uniform, lacking double-blind, randomized, placebo-controlled clinical studies. Most scholars believe that surgery is the best treatment [3, 4]. There is also disagreement among researchers about the effectiveness of drug therapy after surgery. Gebitekin et al. [6] applied pneumonostomy and myoplasty to treat complex aspergilloma, and orally administered itraconazole two weeks before surgery and three months after surgery. The results showed that aspergillomas were completely removed, without death and few postoperative complications. However, in the study of Sagan et al. [7], postoperative adjuvant drug therapy did not improve the prognosis. Because it is difficult for systemic antifungal agents to penetrate the cavity of aspergillus globule, percutaneous or bronchoscopic injection of antifungal agents may be attempted in patients with contraindications or refusal to undergo surgery. Giron et al. [8] intraperitoneally injected amphoricin B into 40 patients with aspergilloma that were not suitable for surgery, and followed up 6 to 28 months after treatment. The results showed that 26 cases of aspergilloma disappeared and the serum test turned negative. Kravitz et al. [9] treated 20 cases of aspergillus hemoptysis with CT-guided percutaneous catheter injection of amphotericin B, and the results showed that hemoptysis symptoms in 17 cases (85%) were effectively controlled in the short term. But all of these studies were local treatment alone, with a varied duration of treatment. There are also some reports showed that intracavitary injection of amphotericin B is ineffective in the treatment of aspergilloma [10].

In general, more evidence-based medical evidence is needed to explore whether antifungal agents should be used after aspergilloma surgery, the appropriate course of treatment and the safety of drugs. There are both advantages and disadvantages of antifungal therapy. The disadvantages are the high expense of antifungal drugs, the heavy economic burden on patients, and the high risk of liver and kidney function damage caused by antifungal drugs. The advantage of antifungal therapy is that it may reduce the risk of aspergillus recurrence. The advantage of non-antifungal therapy is to reduce the overtreatment in some cases and reduce the economic burden of patients. But its disadvantage is that the risk of fungal infection recurrence may be increased. Therefore, more clinical studies are needed to provide evidence-based medical evidence for the following issues: (1) Further analysis is needed to determine whether anti-fungal treatment after aspergilloma bulbectomy has an impact on recurrence of aspergillus infection. (2) Duration and safety of antifungal therapy.

This study was designed to evaluate the difference in recurrence risk and safety of antifungal agents in patients with pathologically confirmed aspergilloma after surgery in a single-center, randomized, parallel grouping, prospective, 2-year clinical study. Through regular supervision record of aspergilloma recurrence, the quality of life, lung function and safety evaluation of fungi treatment, to explore the most suitable treatment for aspergilloma, to improve the quality of life of patients with more advantages, reduce the recurrence of aspergillus infection, improve the prognosis of patients with aspergilloma eventually. The study results will provide high-quality evidence-based medical evidence for the formulation, revision and optimization of international and domestic clinical guidelines and expert consensus on chronic aspergillus lung disease, effectively improve the clinical treatment effect, and form the latest concept of diagnosis and treatment of aspergilloma.

## Data Availability

The datasets used and analysed during the current study will be available from the corresponding author on reasonable request.

## Abbreviations

RR: Relative risk
CI: Confidence interval
AST: Aspartate aminotransferase
ALT: Alanine transaminase
AIDS: Acquired immune deficiency syndrome
CRF: Case report form
G-test: 1,3-β-D glucan detection
GM test: Galactose mannitolglycan antigen detection
Ig: Immune globulin
CT: Computerized tomography
SGRQ: St. George's Respiratory Questionnaire
